# Numerical Simulation of Airway Dimension Effects on Airflow Patterns and Odorant Deposition Patterns in the Rat Nasal Cavity

**DOI:** 10.1371/journal.pone.0077570

**Published:** 2013-10-28

**Authors:** Zehong Wei, Zhixiang Xu, Bo Li, Fuqiang Xu

**Affiliations:** 1 Division of Biomedical Photonics, Wuhan National Laboratory of Optoelectronics, Wuhan, China; 2 State Key Laboratory of Magnetic Resonance, Atomic and Molecular Physics, Wuhan Institute of Physics and Mathematics, Chinese Academy of Sciences, Wuhan, China; University of Zurich, Switzerland

## Abstract

The sense of smell is largely dependent on the airflow and odorant transport in the nasal cavity, which in turn depends on the anatomical structure of the nose. In order to evaluate the effect of airway dimension on rat nasal airflow patterns and odorant deposition patterns, we constructed two 3-dimensional, anatomically accurate models of the left nasal cavity of a Sprague-Dawley rat: one was based on high-resolution MRI images with relatively narrow airways and the other was based on artificially-widening airways of the MRI images by referencing the section images with relatively wide airways. Airflow and odorant transport, in the two models, were determined using the method of computational fluid dynamics with finite volume method. The results demonstrated that an increase of 34 µm in nasal airway dimension significantly decreased the average velocity in the whole nasal cavity by about 10% and in the olfactory region by about 12% and increased the volumetric flow into the olfactory region by about 3%. Odorant deposition was affected to a larger extent, especially in the olfactory region, where the maximum odorant deposition difference reached one order of magnitude. The results suggest that a more accurate nasal cavity model is necessary in order to more precisely study the olfactory function of the nose when using the rat.

## Introduction

Respiration and olfaction are two important physiological functions of the nose. These functions are strongly dependent on patterns of airflow [1]–[5] and odorant deposition [6]–[10] in the nasal cavity. Therefore, determining how these patterns are affected by intrinsic and extrinsic factors is of great importance.

Airflow patterns in the nasal cavities have been investigated *in* *vitro* by a number of researchers using cast molds. Quantitative measurements using dye-streakline [11]–[12], miniature pitot tube [13], radioactive tracer [14] and thermistor probes [15]–[16] were conducted using these molds. However, these methods have some shortcomings, such as spatial resolution and measurement accuracy and they can be time consuming. *In vivo* approaches, such as rhinomanometry and acoustic rhinometry, can determine changes in overall nasal airflow, resistance and cross sectional areas [1], [17]–[19], but are not able to show sufficient details of dynamic airflow through the nasal cavity. However, these weaknesses can be overcome by adopting modern numerical simulation technology.

With the rapid development of computer power, the use of numerical simulation technology has been increasing in biological fields. From micro cells [20]–[24] to macro systems [25]–[27], the role of numerical simulation is becoming increasingly important. Specifically, computational fluid dynamics (CFD) simulation is widely used to explore the physiological functions of the nasal cavity.

So far, respiration-related airflow and odorant transport in the nasal cavity have been studied in several kinds of mammals, such as humans [28]–[35], monkeys [36], dogs [8], [37], rabbits [38], and rats [39]–[45]. The intranasal flow patterns [28]–[34], [36]–[40] or odorant deposition patterns [8]–[10], [35], [41]–[45] have been determined using CFD simulations. In odorant transport simulations, a quasi-steady equilibrium process has generally, and reasonably, been assumed, for simplicity [8]–[10], [35], [46]. It has been reported that differences in nasal morphology [47]–[49], or relatively small changes at specific anatomical locations of the nasal cavity [2]–[5], [50], even those on a micrometer scale [51], may induce large changes in nasal airflow in the humans. Since odorant deposition patterns in the nasal cavity depend largely on nasal airflow patterns [8]–[10], [42]–[46], odorant transport and deposition are also affected, on some level, by variations in nasal airway dimensions [9].

The nasal airway boundary is covered by a mucous layer, which has been described as a superficial watery layer and has an estimated thickness of 5–30 µm among different individuals [52]–[53]. Three-dimensional (3D) rat nasal cavity models have been commonly used in CFD simulations and are reconstructed from 2-dimensional (2D) section images [10], [39]–[45]. The airways of these models are wider than the real nose due to nasal tissue dehydration and mucous loss. However, this can be avoided by using 2D images that are acquired from MRI scans of living animals.

Rats are widely used for studying the sense of smell [6], [10], [54], which is a very important area of neuroscience. However, the influence of variations in airway dimensions on airflow and odorant deposition patterns in the rat nasal cavity have not been explored thus far. The current study used the CFD simulation method in two models of the rat nasal cavity to evaluate the differences in nasal airflow and odorant deposition patterns caused by airway dimension variations: one model, based on MRI images, was reconstructed with narrow airways and the other model, with wide airways, was reconstructed from artificially-widening airways of the MRI images by referencing the section images. The results demonstrated that a small change in the airway dimension could significantly change the airflow and odorant deposition patterns in the rat nasal cavity, especially in the post-dorsal olfactory epithelial (OE) region. These results suggest that, in order to study olfaction more accurately using the rat model, a more accurate nasal cavity model is necessary.

## Materials and Methods

### Animal

Ten-week-old male Sprague-Dawley rat was purchased from Wuhan University Animal Experiment Center, Wuhan, China.

### Ethics Statement

The animal experiments were carried out in strict accordance with the protocols approved by the Animal Ethics Committee at the Wuhan Institute of Physics and Mathematics, Chinese Academy of Sciences (SYXK(E)2009-0051, No. 00011018). All efforts were made to minimize animal suffering.

### Image Acquisition

**Figure 1:**
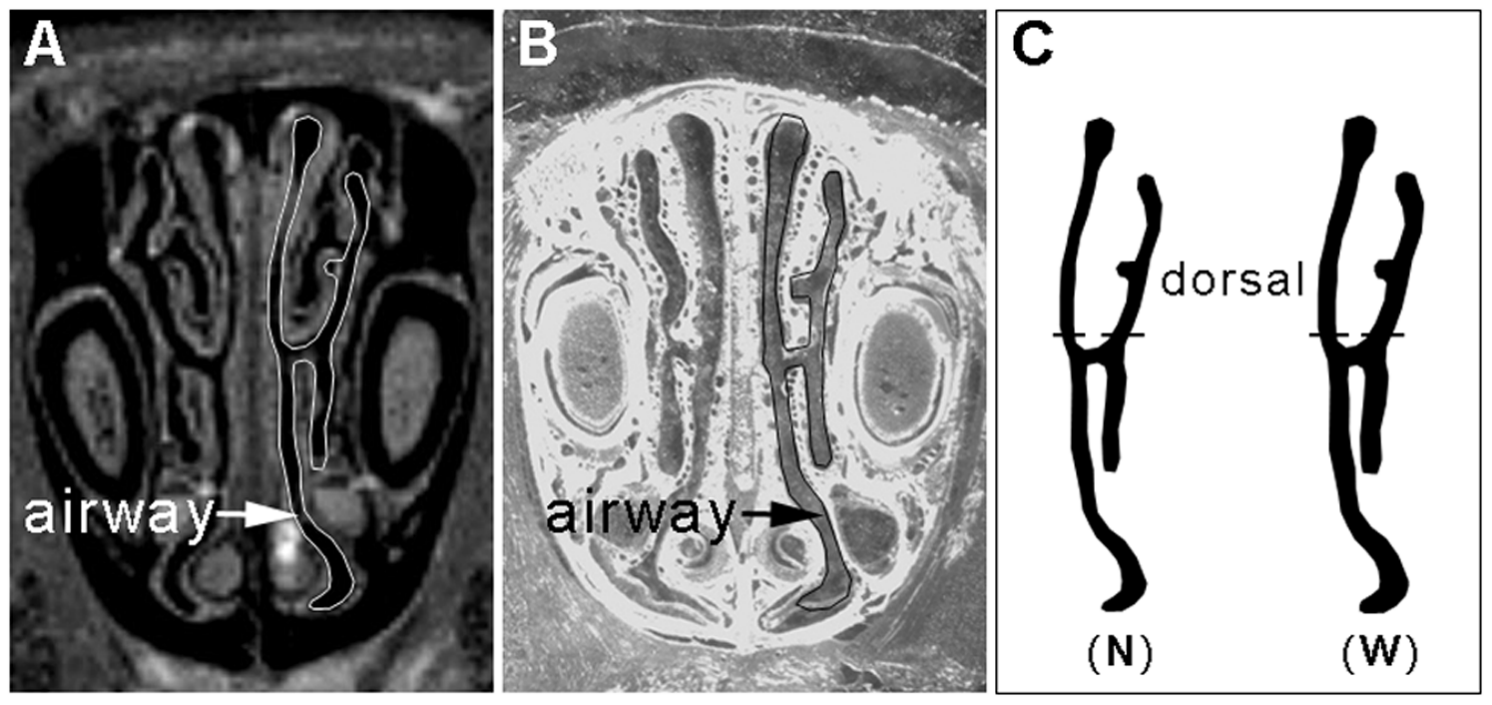
Rat nasal coronal image. (A) MRI. (B) Tissue section image. (C) Segmented left nasal airway binary image; the black area is airway. N: airway from MRI; W: artificially-widening airways of the MRI images by referencing the section images.

**Figure 2:**
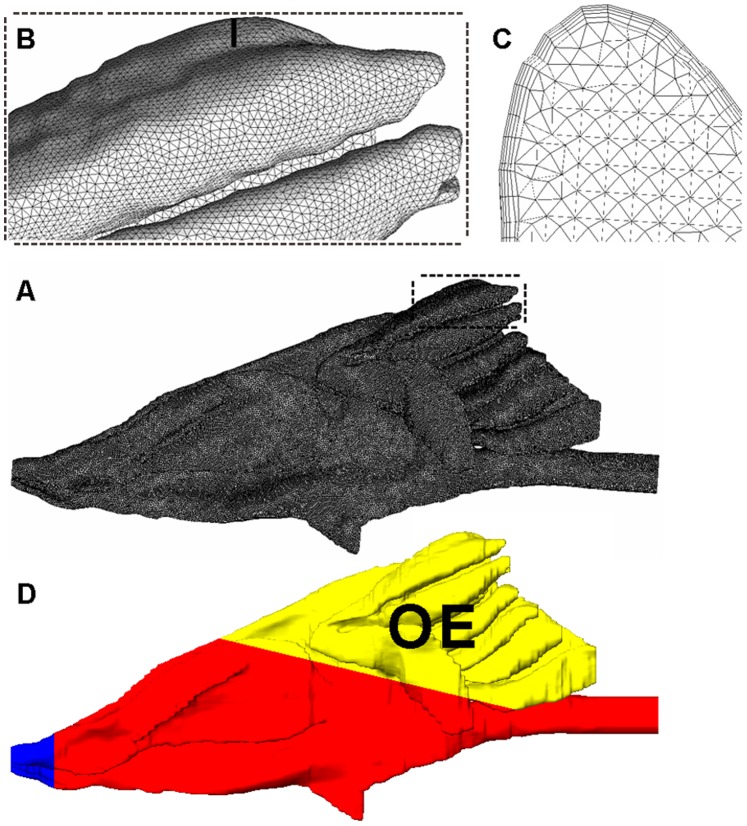
The reconstructed 3D rat nasal model and three marked parts with grids. (A) Sagittal view of the reconstructed 3D model of the left side rat nasal cavity with the global grids. The anterior is external naris, the posterior is nasopharynx and the post-dorsal part is the OE. (B) Grid of a post-dorsal region located at the area surrounded by the rectangle shown in A. (C) A coronal surface grid located at the black stripe region of B. (D) Three parts of the rat nasal model. Blue = squamous epithelium; Red = respiratory epithelium; Yellow = OE.

The rat was anesthetized with 2% halothane, placed on a water heated animal bed and the head was placed in a head holder to minimize body movement. Temperature and respiration were recorded using an animal monitoring device. A series of 100 2D MSME T1-weighted coronal images (Figure 1A), covering the nasal cavity from the external naris to the pharynx (Figure 2A), were acquired using a Bruker 7.0 T MRI scanner (Bruker, USA) with a rat head coil. The imaging parameters were as follows: field of view = 25.6×25.6×30 mm, image dimension = 256×256×100 pixels, in plane resolution = 100×100 µm, slice thickness = 300 µm, relaxation delay = 3000 ms, echo time = 15 ms, number of average = 16 and total time ≈ 3.5 hours.

Following MRI scanning, the rat was euthanized by an overdose of intraperitoneally injected urethane (1.4 g/kg) and then transcardially perfused with saline, followed by 4% paraformaldehyde (PFA) in PBS. The brain was post-fixed in PFA overnight and cryoprotected in 30% sucrose solution. The tissue was sectioned at 25 µm with a freezing microtome (LEICA CM1850, Germany). A series of 100 2D images corresponding to the previous MRI images, were then selected and photographed (Figure 1B). The airway in the section image appeared to be somewhat wider than in the MRI image due to tissue shrinkage (Figure 1A and1B).

### Model Construction and Grid Generation

**Figure 3:**
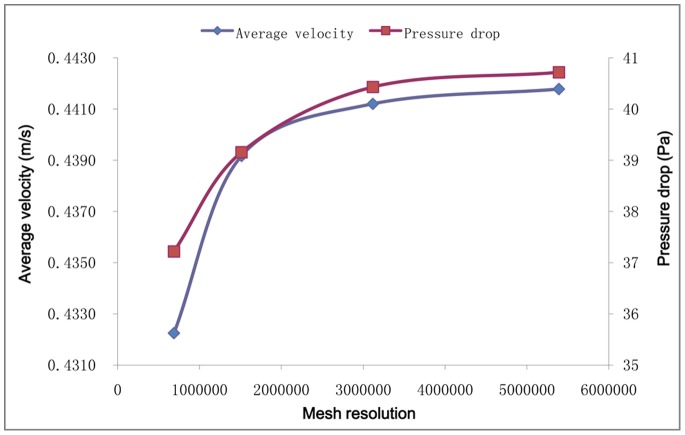
Average velocity and pressure drop throughout NCMn according to different mesh resolutions.

The pixels and document size of the MRI images were adjusted to 12800 × 12800 pixels and 256 × 256 mm by re-sampling the image pixels and adjusting the image document size in Photoshop CS5 (Adobe systems Inc., USA), which made an artificially-widening operation of the airways, and then a model reconstruction process in the next section can be achieved (in effect, the adjusting operation transformed the operating precision in the images from 100 µm to 2 µm). The nasal airway was then segmented from the tissue in the MRI images in order to obtain a series of binary images of the left nasal airways (Figure 1C_N_). The series of binary images of the nasal airway was then imported into AMIRA 5.3.3 (VSG Inc., USA) software for 3D reconstruction of the rat nasal cavity. Through volume rendering methods and smooth operations, a 3D model of the left rat nasal cavity (NCMn) was constructed (Figure 2A) and exported in.stl format to be used by ANSYS ICEM CFD 13.0 (ANSYS Inc., USA) for grid meshing. The tetrahedron/mixed grids were adopted to mesh the model, with five-layer prism layers generated near the boundary surface (Figure 2D) to capture the near wall changes in velocity and odorant concentration. Four meshed models, with grid numbers 687796, 1513583, 3118236 and 5391077, were used for the grid independence test. Results for average velocity and pressure drop at flow rate of 128 mL/min, throughout the nasal cavity, converged as the grid number approached 3118236 (Figure 3). Therefore, the model with 3118236 computational elements was used in this study. The grid parameters for NCMn are listed in Table 1.

**Table 1:** Grid information and part information of the two models.

	NCMn	NCMw
Entire surface (m^2^)	9.14×10^−04^	9.22×10^−04^
Squamous epithelium (m^2^)	1.50×10^−05^	1.52×10^−05^
Respiratory epithelium (m^2^)	3.68×10^−04^	3.72×10^−04^
OE (m^2^)	5.31×10^−04^	5.35×10^−04^
Volume (m^3^)	1.53×10^−07^	1.71×10^−07^
Elements	3118236	3374313
Nodes	1050595	1098601

NCMn: nasal cavity model with narrow airway; NCMw: nasal cavity model with wide airway.

Another nasal cavity model, with relatively wider airways (NCMw), was reconstructed from artificially-widening airways of the MRI images uniformly by 34 µm by referencing the section images, using the above method. The grid parameters of NCMw that correspond to NCMn are listed in Table 1. The results for average velocity and pressure drop (only somewhat lower than in NCMn, therefore was not shown), throughout NCMw in the grid independence test, converged as the grid number approached 3374313.

In order to evaluate the difference in odorant deposition patterns between the two models, especially in the OE region, both models were simplified into three parts, according to previous studies: the OE, the respiratory epithelium (the post-ventral nasopharynx tube, whose function is equal to respiratory epithelium in this study, is categorized into respiratory part for simplicity) and the squamous epithelium [42], [54] (Figure 2D). The OE and respiratory epithelium are both coated by mucus, which can absorb odorant molecules. The squamous epithelium histologically resembles skin and is not coated by mucus thus it does not absorb odorant. The parameters of the two models are listed in Table 1.

Three odorants with different solubilities (carvone, high solubility; amyl acetate, intermediate solubility; octane, low solubility) were used to simulate their depositions, in all regions of the two models, under three physiological inspiratory flow rates. The physiochemical properties of these odorants are presented in Table 2.

**Table 2:** Physiochemical properties of odorants at 25°C and 1 atm.

Odorant	*D_a_* (m^2^/s)	*D_m_* (m^2^/s)	*β*
Carvone	6.2×10^−6^	6.9×10^−10^	1.3×10^−4^
Amyl acetate	6.7×10^−6^	7.8×10^−10^	2.5×10^−3^
Octane	6.0×10^−6^	7.4×10^−10^	0.48

*D_a_*: the diffusivity of the odorant in the air; *D_m_*: the diffusivity of the odorant in the mucus; *β*: the odorant equilibrium partition coefficient between air and mucus. All the parameters used here are from a previous paper [35].

### Governing Equations and Boundary Conditions

For the steady-state flow of air and transport of odorant in the model, the governing equations used were the Navier-Stokes, continuity and the convective-diffusion equations. The first 2 sets of equations (1 and 2) are known as flow equations and represent airflow. The last equation (3) is known as the convective–diffusion equation and describes odorant transport
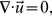
(1)

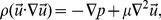
(2)

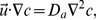
(3)


Where 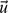 is the 3D velocity vector (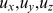) in air and *c* is the nondimensionalized odorant concentration in the nasal cavity. *D_a_* is the odorant diffusivity in air, and 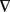 and 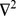 are the gradient and Laplace operators, respectively. In the present study, the CFD software package ANSYS FLUENT 13.0 (ANSYS Inc., USA) was used to solve these equations through a finite volume method.

For the boundary conditions of the flow equations (1 and 2), the walls of the model were assumed to be rigid and the no slip air velocity was applied at the walls. The velocity-inlet boundary (which specifies a uniform flow at the inlet) was applied at the external naris (inlet) and the outflow boundary (which assumes zero normal of the fluid variable at the outlet) was applied at the nasopharynx (outlet). Three physiological respiratory flow rates, 128, 256 and 512 mL/min [10], [39]–[40], [55], were used to calculate the velocity field. At these flow rates, the Reynolds numbers at the inlet are all less than 600 and therefore it is reasonable to adopt the laminar flow model [56]. Unsteadiness in the flow field can be disregarded because the Strouhal number is much less than unity, and thus a quasi-steady boundary layer in the nasal airways can be established [35], [40], [56]–[58].

**Figure 4:**
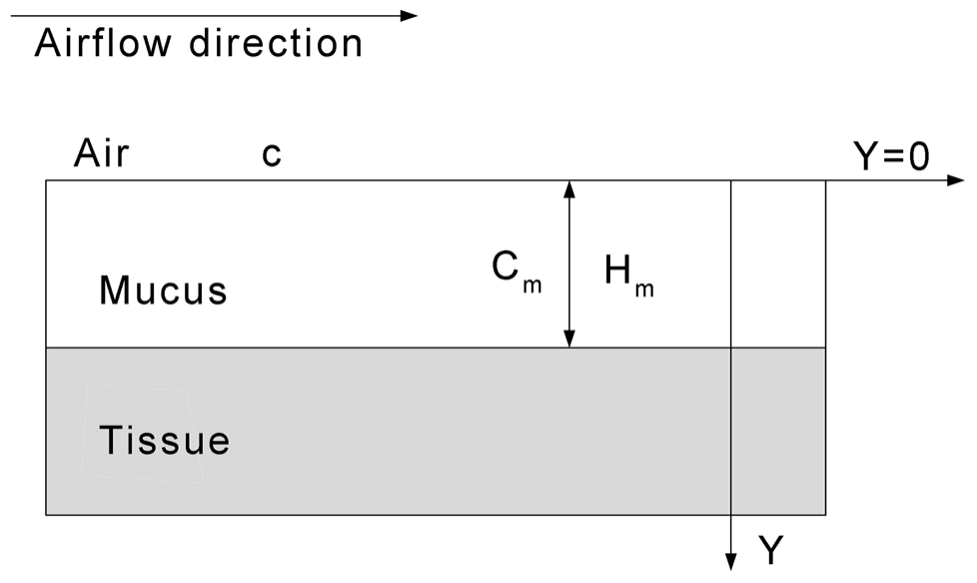
A diagram of the mass transfer boundary condition at the air-mucus interface [35].

For the boundary condition of the convective-diffusion equation (3), a mass transfer boundary condition was applied at the walls (the interface between the air and mucus), as previously described [35], [46], to simulate odorant deposition during inspiration (Figure 4).

The mass transfer boundary condition at the wall includes odorant molecules from inhaled air that are absorbed into the olfactory mucus, diffused across the mucus layer, and finally removed by the submucosal blood flow. The odorant concentration was set so that it was equal to zero (*c = *0) at the mucus/tissue interface. The non-dimensional steady-state mass transfer boundary condition (a quasi-steady equilibrium transport process was reasonably assumed [8]–[10], [35], [46]) at the wall is given by
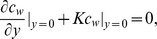
where *c_w_* is odorant concentration at the air-mucus interface and *y* is the coordinate in the direction perpendicular to the air-mucus interface. The non-dimensional parameter *K* is given by 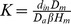, where *d_in_* is the hydraulic diameter of the inlet and *D_a_* and *D_m_* are the diffusivity of the inhaled odorant in the air and mucus, respectively. *β* is the odorant equilibrium partition coefficient between air and mucus and *H_m_* is the thickness of the mucus layer, which was assumed to be uniform at 30 µm [52]–[53]. All odorant parameters used here are exactly those used in a previously published paper [35] (Table 2). The *β* values are based on results from experimental measurements done on bullfrogs [59]–[60].

The anterior squamous epithelium of the rat nose cannot absorb odorants, so the zero-wall mass flux boundary condition was applied here. The uniform dimensionless concentration boundary condition of *c* = 1.0 was applied at the inlet and the outflow boundary condition was applied at the outlet.

### Interpolation and Solution Methods

The ANSYS FLUENT 13.0 (ANSYS Inc., USA) pressure based solver was used to numerically solve equations with the boundary conditions noted above. The SIMPLE algorithm was chosen as the pressure and velocity couple method. The interpolation scheme used for pressure was the second order and the interpolation scheme used for velocity and species was the second order upwind. The computations were carried out on a PC with the WIN7 operating system. A norm of nodal concentration differences between iterations of less than 10^−6^ was used as the convergence criterion for the termination of concentration iterations; other variables were set as less than 10^−4^. That is,
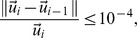


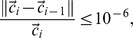
where 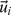 is velocity solution vector, *c_i_* is the concentration solution at iteration *i* and ||*|| is the root mean square norm summed over all of the nodes of the model grid. Conservation of mass in the model was checked for in the final solution; the difference between the odorant mass entering the inlet and exiting the outlet was equal to the total deposition on the nasal mucosal wall. Other flow variables, such as the average concentrations near the respiratory wall and olfactory wall, were also defined to monitor the convergence of the iterative process.

### Computation of Deposition dose on the Nasal Wall

When the odorant concentration in the nasal cavity was obtained, the normal component of mass flux (kg/m^2^*s) at any position on the wall surface of the 3D grid was described in the form 
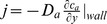
. The total odorant deposition, *J* (kg/s), over a given wall surface was then determined by integrating *j* over the wall surface.

## Results

### Comparison of Average Velocity

Velocity field is the most important parameter used to describe flow pattern. Therefore, the difference in the flow patterns between the two models was primarily evaluated by comparing the velocity, especially the average velocity, which can reflect the difference as a whole.

**Figure 5:**
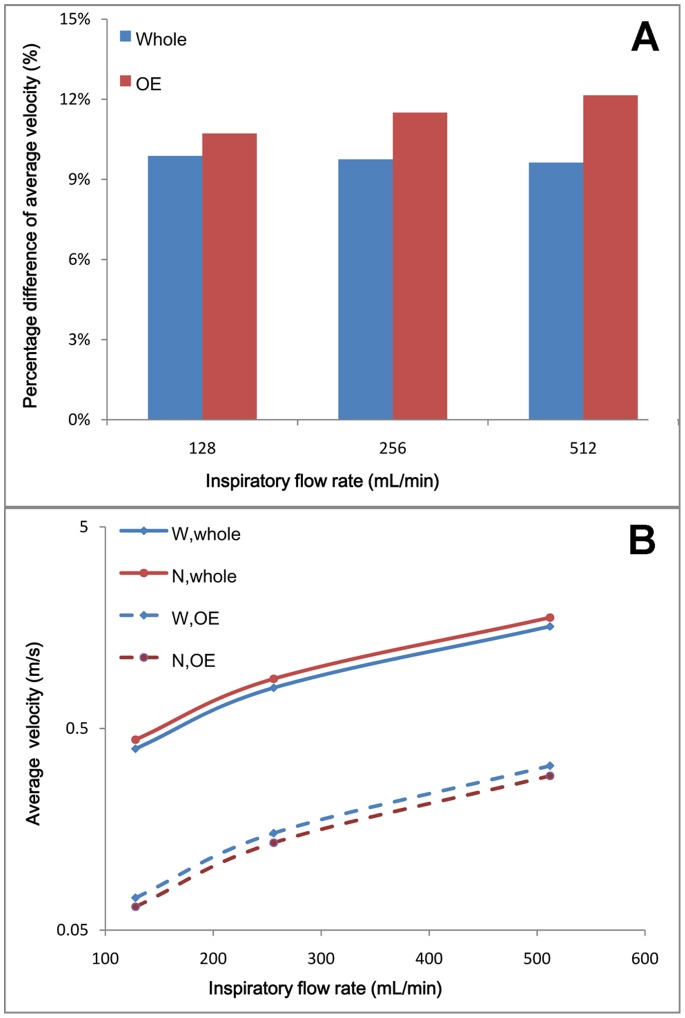
Average velocity and the percentage difference between the two models. (A) Whole: percentage differences of average velocity in the whole model; OE: percentage differences of average velocity in the OE region. (B) W, whole: average velocity in the whole NCMw; N, whole: average velocity in the whole NCMn; W, OE: average velocity in the OE region of NCMw; N, OE: average velocity in the OE region of NCMn.

The computed results showed that, for the whole model, the average velocity in NCMw was about 10% (Figure 5A) lower (Figure 5B) than in NCMn for all three inspiratory flow rates. The whole difference was clear and was independent of the flow rate. However, the situation was reversed in the OE region. That is, the average velocity in NCMw was about 12% (Figure 5A) higher (Figure 5B) than in the NCMn and this difference increased slightly as the flow rate increased.

### Comparison of Flow Distribution

Flow distribution through two selected zones, the “dorsal” part of a coronal section (Figure 1C) in the anterior part of the nose and the whole OE (Figure 2D), was computed to determine the effect of airway dimension on those regions. Volumetric flow throughout a selected section was determined by integrating the velocity profile over the selected section, and the percentage of volumetric flow in a subsection was determined by dividing the volumetric flow of the subsection by total volumetric flow. Percentage of volumetric flow in the “dorsal” part and OE were computed. The data are listed in Table 3.

**Table 3:** Percentage of volumetric flow distribution in the “dorsal” part and OE.

Flow rate (mL/min)		128	256	512
“dorsal” part	NCMn	45.21%	45.34%	45.70%
	NCMw	45.60%	45.81%	46.37%
OE	NCMn	33.75%	34.69%	37.45%
	NCMw	36.62%	37.51%	40.27%

NCMn: nasal cavity model with narrow airway; NCMw: nasal cavity model with wide airway; “dorsal” part: the dorsal part marked in Figure 1C; OE: olfactory epithelium in Figure 2D.

The results showed that, in the “dorsal” part, the percentage difference in the NCMw was a little larger (<0.7%) than in the NCMn, for all flow rates, and increased slightly as the flow rate increased. The differences were larger for the whole OE. Specifically, the percentage difference between NCMw and NCMn was ∼3% at all flow rates.

### Comparison of Odorant Deposition Pattern

Odorant molecules flow into the nasal cavity, are absorbed into the nasal mucus and produce an odorant deposition pattern in the nasal mucus. The difference in the odorant deposition patterns, between the two models, was evaluated.

The amount of odorant deposition on the two nasal cavity walls, and especially the OE, was computed and results are listed in Table 4. The results indicated that the difference in odorant deposition between the two models varied greatly according to the odorant solubility. That is, the more soluble the odorant, the larger the difference in deposition. Moreover, the absolute deposition amount increased as the flow rate increased. Specifically, for odorants deposited on the whole nasal cavity walls, the difference in the deposition amount was relatively small and a maximum percentage difference of 4.93% occurred at 512**mL/min for the most soluble odorant, carvone. However, for odorants deposited on the OE, the difference in the deposition amounts was much more significant. For example, at a flow rate of 128 mL/min, the percentage differences in the deposition amounts for octane, amyl acetate and carvone were 8.26%, 26.18% and 1158.54%, respectively.

**Table 4:** Odorant deposition amount on the nasal cavity wall.

		Whole nasal cavity						OE						
Flow rate (mL/min)		128		256		512		128		256		512		
octane	NCMn		1.95		2.02		2.07		1.09		1.16		1.20	
	NCMw		2.02		2.10		2.11		1.18		1.22		1.24	
amyl acetate	NCMn		84.55		130.11		180.31		7.60		22.07		46.33	
	NCMw		90.39		137.43		188.12		9.59		26.53		52.05	
carvone	NCMn		127.44		249.33		460.60		2.05×10^−2^		6.48×10^−1^		4.34	
	NCMw		121.98		237.63		437.87		2.58×10^−1^		5.14		28.05	

NCMn: nasal cavity model with narrow airway; NCMw: nasal cavity model with wide airway. The unit of the deposition amount is kg/s (×10^−7^).

**Figure 6:**
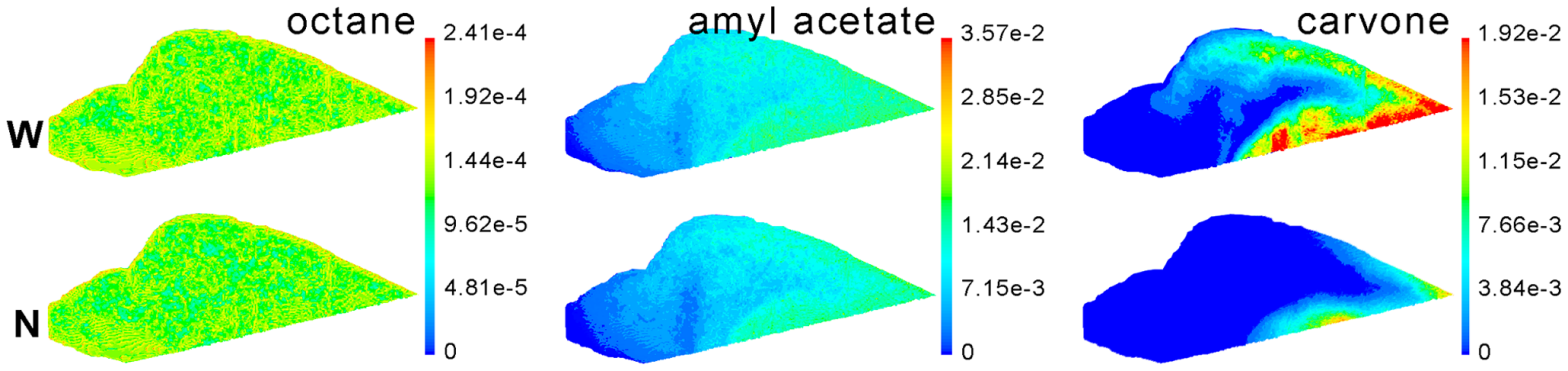
Flux contour of three odorants on the septum of the OE. The unit is kg/m^2^*s. Flow rate is 512 mL/min. Deposition amounts higher than the maximum value of the color map are represented by the most extreme red color in the spectrum. (N) NCMn; (W) NCMw.

**Figure 7:**
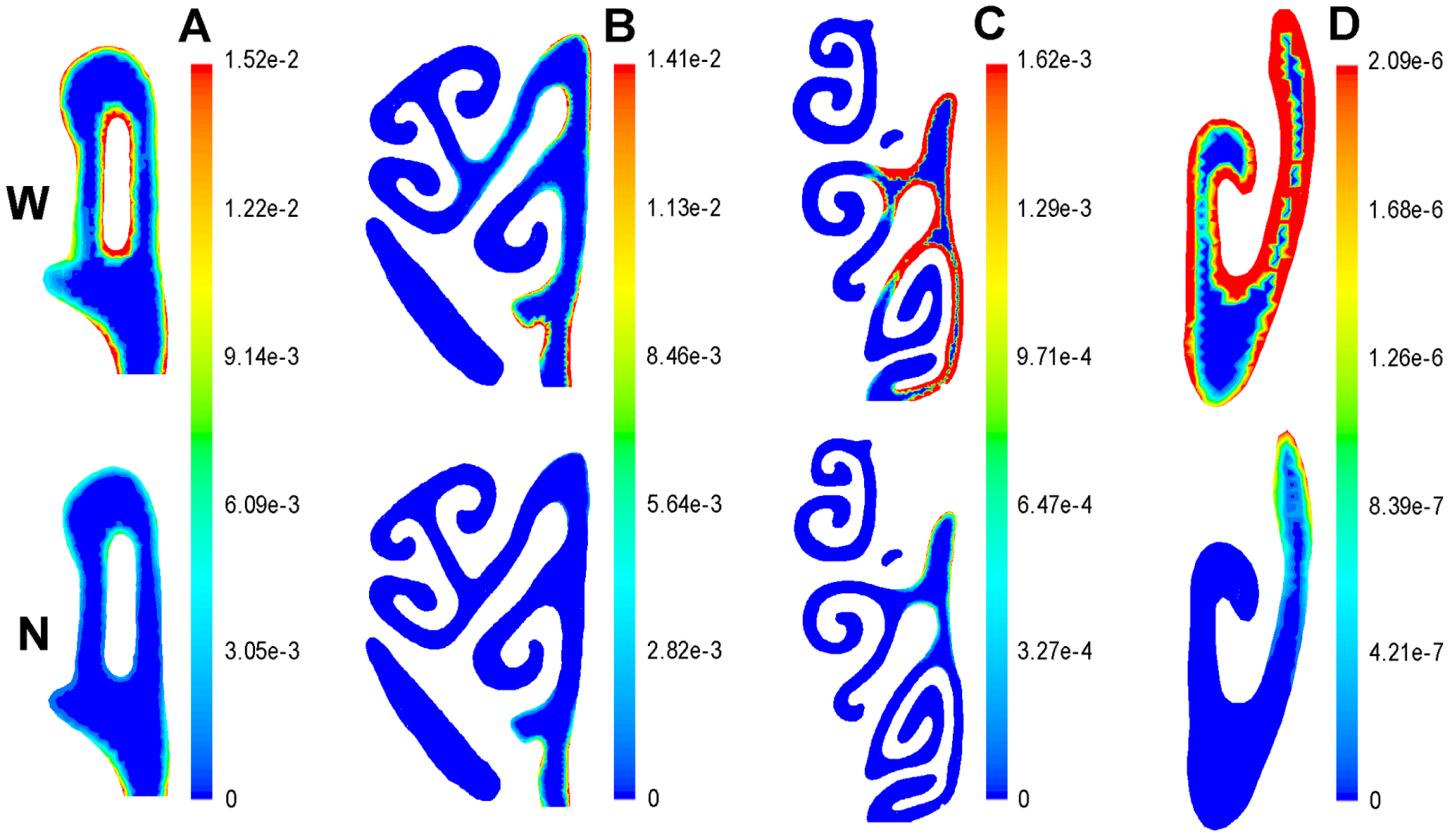
Flux contour of carvone in four coronal sections at OE. These sections were taken at the following distances from the nostril: (A) 14 mm, (B) 18 mm, (C) 22 mm and (D) 26 mm. The unit is kg/m^2^*s. Flow rate was 512 mL/min. Deposition amounts higher than the maximum value of the color map are represented by the most extreme red in the color spectrum. (N) NCMn; (W) NCMw.

To display the odorant deposition pattern and difference intuitively, the odorant flux contour on the septum of the OE is presented (Figure 6). The results indicated that, for the less soluble octane, the odorant deposition pattern between the models was generally the same; for intermediate amyl acetate, the odorant deposition pattern between the models was a little different; but for the highly soluble carvone, there was a very big difference in the odorant deposition pattern between the models. Four coronal sections, located at the OE region, were also selected to more concretely display the distinct difference (Figure 7).

## Discussion

### Model One

The coronal section and the marked “dorsal” part (Figure 1C) in this paper were similar to plane 126 and the marked “DM+DL” part in the study by Yang et al. [40] and also similar to K23 and the marked “DMS+DMN+DL” in the study by Kimbell et al. [39]. The percentage of volumetric flow distributions in the “dorsal” part (∼46%) and the changing value (∼0.4%) with the flow rates found in NCMw in this study are in general agreement with those reported by Yang et al. (∼45% and ∼1.5%, respectively) and Kimbell et al. (∼52% and ∼0.8%, respectively). The different models, used in their respective studies, may account for the small differences in results.

The magnitude of the three amounts of odorant deposition in the whole model, and the changes with the volumetric flow rates, reported in NCMw in this paper (Table 4) are also in general agreement with the previous work of Yang et al. [10]. Any small differences between the results from the present study and those from previous studies could also be due to differences in the models used.

The flux patterns of the three different soluble odorants on the nasal walls (Figure 6 and 7) were generally consistent with the previous work of Yang et al. [10] and Zhao et al. [45]. Similar patterns have also been found by Zhao et al. [9], [45] in the human nose and by Lawson et al. [8] in the dog nose.

### Comparison between Two Models

It is well known that tissue shrinks after fixation and can be significantly distorted [61]–[63]. This shrinkage and distortion can cause models that are based on tissue section images, to deviate from actual anatomical structures. One of the models in the present study, NCMn, was reconstructed using MRI images collected from a live rat, while the other model, NCMw, was reconstructed from the artificially-widening airways of the MRI images by referencing the section images of the same rat, similar to earlier rat nasal models [10], [39]–[45]. This study evaluated the differences in airflow and odorant deposition patterns between the two models in order to provide further reference for the accurate study of olfaction and olfactory neuroscience. A few points to note: first, a NCMw which matches the NCMn appropriately for comparative study could not be constructed directly from the section images, due to a few technical reasons with our current slicing technique such as: the orientation of all sections could not be guaranteed; very hard to have each section avoid of distortions (maybe we could solve it with a more advanced slicing technique and photographing technique in the future). Second, due to the first point and our purpose is to examine the effects of changes in airway dimension on nasal airflow and odorant deposition patterns in a more realistic nasal cavity model built from a live animal, a NCMw with idealized assumption was constructed from artificially-widening airways of the MRI images by referencing the section images (here, an artificially-widening nasal airways of the MRI images uniformly by 34 µm were used to account for potential tissue shrinkage and loss of mucus layer in the section images, based on the following reasons: corresponding positions of the airways, in the two sets of images, were examined. In general, the airways in the section images were found to be uniformly wider around the boundary, across the nasal cavity. The uniform assumptions were used commonly in similar works [8]–[10], [35], [45]). Third, in effect, the NCMw can take the place of the model that based on the section images to some extent and it should not alter the conclusions from the report according to the research purpose. That is, the conclusions drawing from the NCMw in this study can reflect the effect on airflow and odorant deposition patterns, caused by the airway dimension changes that come from the real airway shrink, to a certain degree.

The percentage of the average velocity differed by ∼10% between the two whole models and by ∼12% between the OE regions (Figure 5). This demonstrated that the width of the airway has a strong effect on the airflow pattern in the rat nasal cavity, especially in the OE region. In fact, a very small error (∼34 µm) in the anatomical structure of the model can lead to a significant error in reported nasal flow. Similar results have been reported for nasal structure changes in human nasal cavities, such as inferior turbinate hypertrophy [2], inferior turbinate surgery [5] and nasal bone fracture [50]. One point to note is that, in the OE region, the average velocity in NCMw was higher than in NCMn, which was opposite to what was found in the whole model (Figure 5). One reason could be that the inspiratory volumetric flow rates were specified as the same for the two models at the naris, therefore a relatively wide airway resulted in a relatively low average velocity in the whole nasal cavity, resulting in a lower average velocity in NCMw than in NCMn. However, in the OE region, the airway dimension was much smaller, which lead to increased resistance at the nasal wall. A relatively wide airway would provide a relatively small resistance to airflow, therefore, the average velocity in the NCMw would decrease relatively slowly, and consequently, the average velocity would be higher than in the NCMn. This could lead to a larger volumetric flow and odorant deposition in the OE of the NCMw than in the NCMn (Table 3 and 4). If NCMw is used for study of the olfactory sense, this may suggest that the intensity of the olfactory response is somewhat higher than it actually is.

The volumetric flow through the OE in NCMw was ∼3% higher than in NCMn at all flow rates (Table 3), which was consistent with results from Zhao et al. [9]. In that study, they found that an enlargement in the olfactory slit of the human nose increased the volumetric flow into the olfactory region. In the current study, the ∼3% difference was apparent, though not very large. However, multiplying the total volumetric flow by 3% makes a sizeable volumetric flow difference (3.84, 7.68 and 15.36 mL/min for inspiratory flow rates of 128, 256 and 512 mL/min, respectively), especially at high flow rates. Such a large difference in volumetric odorant flow through the OE region could lead to big differences in odorant deposition, and consequently olfactory response. This difference is partially responsible for the big difference in odorant deposition patterns at the OE region (Table 4, Figure 6 and 7).

Since the airflow pattern largely determines the odorant deposition pattern in the nasal cavity [8]–[10], [42]–[46], the odorant deposition pattern is bound to be affected by airway variations [9]. There were significant differences in the odorant deposition patterns between the two models, with an especially large difference at the OE, where the maximum percentage difference in amounts of deposition reached one order of magnitude (Table 4). An extremely significant difference of the flux contour was also noted (Figure 6 and 7). These results suggest that the accuracy of the nasal cavity model, used for simulation, is very important.

The odorant deposition pattern was affected by three main factors with airway dimensions variation. First, odorant flux was affected to a larger extent in areas of the anatomy that were further post-dorsal. Note that, in the OE region, the further posterior a section was located, the greater the difference in odorant flux at the wall (Figure 7). Secondly, the more soluble the odorant, the more the deposition amount was affected. Carvone, the most soluble odorant, had a one order of magnitude difference in deposition amount (Table 4). Third, the higher the volumetric flow rate, the larger the absolute difference in odorant deposition amount, especially in the OE (Table 4). The three main aspects that had the most impact on the odorant deposition patterns as airway dimensions varied, indicate that the biggest errors will occur in these cases when NCMw is used to study nasal olfaction.

The sense of smell is a very important and interesting area of neuroscience research and the rat has been widely used as an experimental model [6], [10], [54]. Results of this study provide recommendations for a more accurate olfactory research, using the rat. This is particularly essential in a field where numerical simulation technology is widely applied. However, there is one point to clarify that the percentage difference used in the results section is the relative value. The difference in the absolute values of the odorant deposition amounts should be considered comprehensively when examining olfactory senses, since the real odorant amounts deposited to the OE determine the strength of an odorant’s signal to the olfactory receptor neurons.

## Conclusions

The CFD method was used to compute airflow and odorant transport in two nasal cavity models: one had narrow airways and was reconstructed using MRI images and the other had wide airways and was constructed from artificially-widening airways of the MRI images by referencing section images. The differences in the airflow and odorant deposition patterns were compared quantitatively between them. The results demonstrated that a small variation in airway dimension could significantly affect the airflow and odorant deposition patterns in the nasal cavity, especially in the OE region. Our results suggest that, a more accurate model that used for investigating olfaction, when using the rat model, is necessary.
